# Compound flood simulations indicate rising public exposure to sewage-contaminated waters in Waikīkī, Hawai‘i

**DOI:** 10.1038/s41598-026-38225-z

**Published:** 2026-02-18

**Authors:** Kayla Yamamoto, Shellie Habel, Youngjun Son, Tiffany R. Anderson, Chloe Obara, Matthew M. Barbee, Kyrstin L. Fornace, Charles H. Fletcher

**Affiliations:** 1 Department of Earth Sciences, School of Ocean and Earth Science and Technology, University of Hawai‘i at Mānoa, POST Building, 1680 East-West Road, Honolulu, HI 96822 USA; 2 Pacific Northwest National Laboratory, 3200 Innovation Blvd, Richland, WA 99354 USA; 3 University of Hawai‘i at Mānoa, Sea Level Center, and CIMAR, 1680 East-West Road, Honolulu, HI 96822 USA

**Keywords:** Compound flooding, Contamination, Sea-level rise, WRF-hydro-CUFA, Drainage failure, Climate change, Climate sciences, Environmental sciences, Hydrology, Natural hazards, Ocean sciences

## Abstract

Waikīkī, Hawai‘i serves as a valuable case study for flood vulnerability due to its low elevation, present-day exposure to multiple flood sources, and role as the state’s economic engine. This study applies the high-resolution, open-source WRF-Hydro-CUFA (Coastal Urban Flood Application) model to simulate compound flood interactions among pluvial, fluvial, coastal, storm drain-driven, and subsurface processes, and to evaluate how flood characteristics in Waikīkī evolve under projected sea-level rise (SLR). Simulated water levels in an estuarine drainage canal, serving as the primary outlet for gravity-flow drainage from Waikīkī, show strong agreement with observations (*r* ≥ *0.935, R*^*2*^ ≥ *0.874*), accurately reproducing flooding observed during recent 5-year and 50-year storm events. Simulations incorporating SLR demonstrate a shift in dominant flood drivers as sea levels rise. In addition, results identify critical thresholds at which elevated estuarine tailwater conditions cause widespread drainage backflow, eventually occurring in the absence of rainfall. These findings suggest that tidally driven flooding will progressively dominate over rainfall-driven events, increasing public exposure to highly contaminated canal and groundwater sources. The results underscore the urgent need to integrate water quality issues and drainage failure into coastal flood management strategies for low-lying urban environments, particularly those reliant on polluted estuarine waterways for stormwater management.

**Supplementary Information:**

The online version contains supplementary material available at 10.1038/s41598-026-38225-z.

## Introduction

Climate change increases global flood risks by raising sea levels, shifting precipitation patterns, and increasing the frequency and intensity of extreme weather events^1–3^. This is particularly evident in low-lying coastal areas, where flood impacts are exacerbated by the interaction of multiple flood drivers through a process commonly referred to as compound flooding. Future climate projections indicate global sea-level rise (SLR) will exponentially increase the frequency of coastal flooding, suggesting that 90% of U.S. coastal regions will experience a daily exceedance of present-day 50-year extreme water levels (i.e., 2% annual chance of exceedance) by the end of the twenty-first century^4^. Moreover, without proper implementation of coastal adaptation measures, over half of the global population will be at risk of flooding^1^.

Despite a growing awareness of flood hazards, populations have continued to settle and expand into present-day flood zones^5–7^. Urban development in these areas further increases flood exposure by introducing impervious surfaces, such as buildings and sidewalks, into the environment. These surfaces significantly reduce water infiltration into the ground, and in-turn increase surface runoff and overall flood severity. Further, coastal gravity-based drainage systems are increasingly experiencing failure due to inadequate infrastructure and outdated design standards, compounded by increasing extreme rainfall and elevated receiving waters^8–11^. High tailwater conditions occur when water levels downstream of a drainage outlet or canal are elevated, which can reverse gravity-driven flow and lead to inland flooding from storm drain backflow. This phenomenon amplifies flood risks in coastal-urban environments, where drainage systems often struggle to effectively manage increasing volumes of floodwater. These complex dynamics within coastal-urban systems highlight the multifaceted nature of compound flooding and the need for an integrated understanding of how coastal, pluvial, and infrastructure-driven processes interact. Such insight is essential for effective risk assessment and resilience planning, particularly in increasingly vulnerable coastal communities^8,12,13^. Given that under-resourced and economically disadvantaged areas often face disproportionately higher flood vulnerability, it is crucial to support the development and implementation of open-source, community models that can guide local planning and decision-making^7,14,15^. Recent advances in climate research identify the key drivers of urban flooding to be precipitation, topography, land cover, infrastructure, and SLR^16^. Therefore, adopting a flood modeling framework that incorporates these drivers is vital for producing robust flood analysis and for informing equitable, evidence-based resilience strategies.

A review by Xu et al. (2020) summarized the growing range of compound flood modeling approaches developed for coastal areas^17^. These include probabilistic and process-based methods that represent different levels of physical complexity. Statistical copula-based models estimate joint flood probabilities^18,19^, while process-based hydrodynamic models dynamically resolve water levels under interacting flood drivers^20,21^. More recently, coupled hydrologic-hydrodynamic frameworks have been introduced to capture feedback between runoff, drainage, and coastal processes^22,23^. For example, Rosenzweig et al. (2025) applied the Coupled Ocean–Atmosphere-Wave-Sediment Transport (COAWST) system to Jamaica Bay, New York, to simulate pluvial and coastal flooding during Post-Tropical Cyclone Ida, capturing interactions among extreme rainfall, storm surge, and tidal forcing^24^. Liu et al. (2025) developed a fully coupled land-river-ocean coupled modeling system for the Pearl River Delta, China, to simulate compound flooding driven by storm surge, tides, river discharge, and precipitation during multiple typhoon events^25^. Such efforts, along with other recent studies, reflect significant progress toward more physically integrated representations of compound flood processes across rainfall, riverine, and coastal domains. However, most of these approaches simplify or omit stormwater drainage processes, limiting their ability to capture backflow dynamics within urban infrastructure. Building upon recent advancements, Son et al. (2023) developed the coupled, hydrology-stormwater hydraulics WRF-Hydro-CUFA (Coastal Urban Flood Application)^26^ modeling framework by expanding the open-source, distributed WRF-Hydro^27^ model to include a dynamic coastal boundary and stormwater drainage modeling, enabling hyper-resolution simulations of compound flooding in coastal urban environments.

Waikīkī, a low-lying district in Honolulu, Hawai‘i, has been the focus of multiple studies on SLR-induced compound flooding. In a study by Habel et al. (2020), discrete flood components, including groundwater emergence, storm drain backflow, and coastal hydrostatic flooding, were individually simulated to identify locations vulnerable to multiple flood sources^28^. The study applies a simplified approach to identify storm drain backflow by locating drainage inlets exceeded by elevated sea level. While useful for planning, the methodology ignores interaction between flood drivers and only considers conditions absent of precipitation. A subsequent and more advanced drainage modeling study by Obara et al. (2025) used PCSWMM to simulate storm drain functionality in Waikīkī, incorporating rainfall and tailwater forcing, validated with observations collected throughout the drainage conduit system^29,30^. The study revealed that as SLR progresses, miles of storm drainage in Waikīkī will increasingly fail, leading to escalating reverse flow from the adjacent contaminated waterway. While the study was able to identify locations of progressively failing drainage, it did not account for interaction between flood sources beyond their direct influence on drainage. Additionally, it lacked the capability to spatially characterize flood extents and depths generated by compound flood sources. The present study improves upon these prior efforts by explicitly modeling the interactions among the major flood drivers within a single, fully coupled modeling framework to quantify inundation depths and extents. This integrated approach enables a process-based evaluation of how inundation sources interact to drive compound flooding in Waikīkī, an advancement beyond prior studies mentioned.

**Fig. 1:**
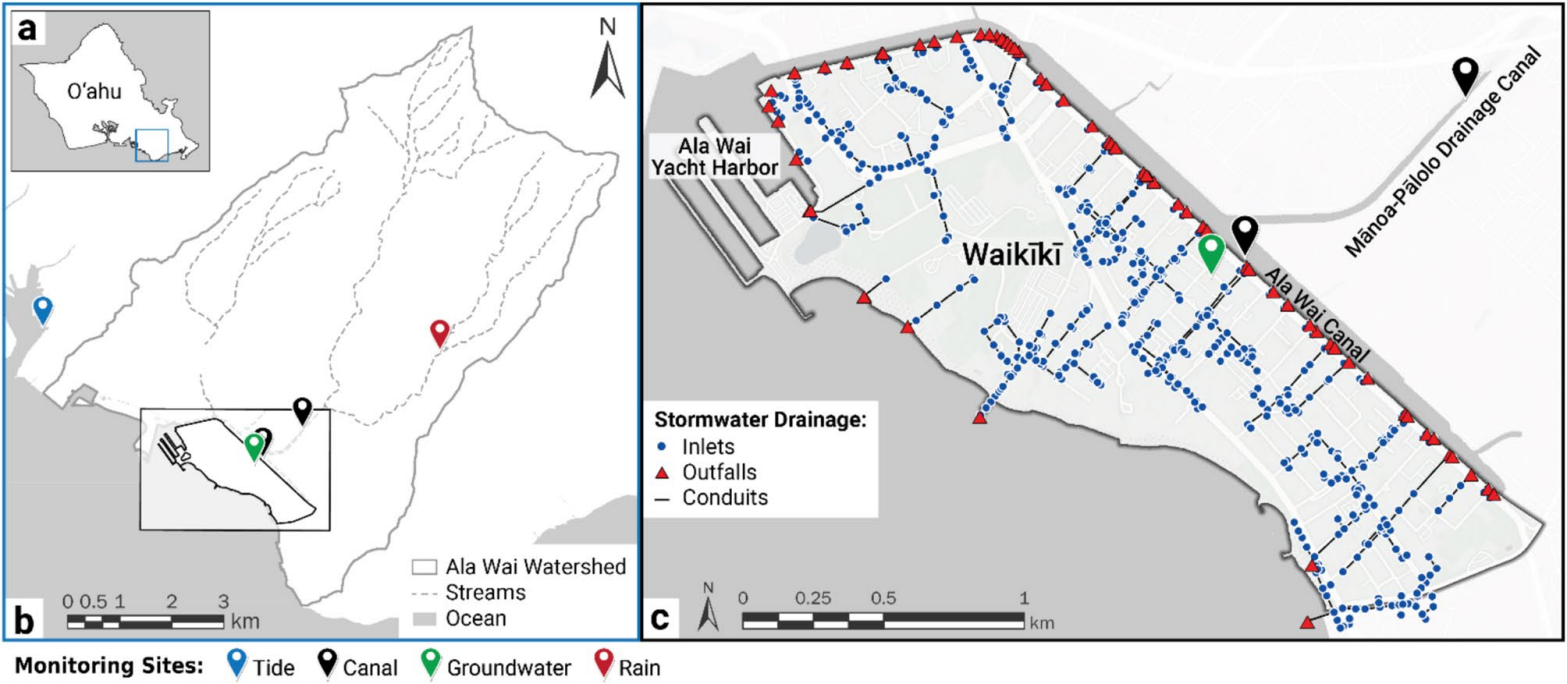
**a)** Location map; **b)** modeling domain inclusive of the entire watershed; and **c)** Waikīkī study area. The Waikīkī stormwater drainage system is configured based on a recent study that simulated Waikīkī drainage dynamics^29^. Monitoring sites described in the present study include the NOAA operated Honolulu tide gauge, a water level transducer placed in the Ala Wai Canal that captures tailwater conditions of the waterway, a USGS stream gauge that captures water levels in the Mānoa-Pālolo drainage canal, a groundwater monitoring well located in central Waikīkī, and a rain gauge operated by NOAA located at the Pālolo Fire Station.

**Fig. 2:**
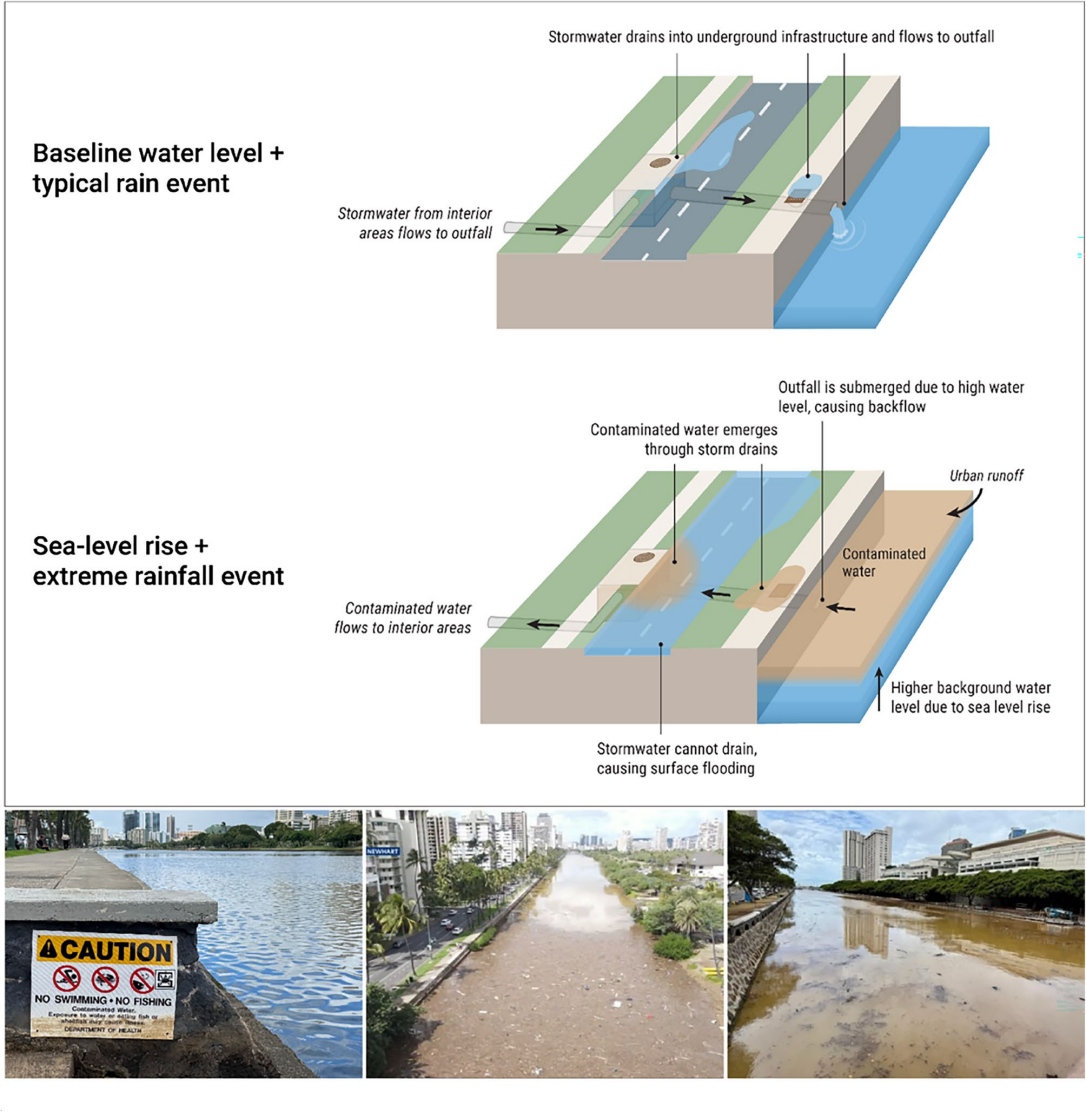
**(Top):** Illustration depicting processes that can cause contaminated backflow through drainage conduits during compound flood events. When tailwater conditions of receiving waters rise above drainage outfalls and inlets, reverse flow can occur. This effect is driven by the combined influence of precipitation and tides on estuarine waterways and is further exacerbated by rising sea levels, which progressively elevate baseline tailwater conditions. **(Bottom):** Images from local sources highlighting the extent to which the Ala Wai Canal is contaminated with sewage and debris. Left: Photograph by Ku ‘upilialoha Veerman; Center: Photograph by Twain Newhart; Right: Photograph by Michael Cain.

To enhance the understanding of compound flood dynamics, the present study employs WRF-Hydro-CUFA to simulate the compound effects of pluvial, fluvial, coastal, storm drain-driven, and subsurface urban flood processes in Waikīkī, Hawai‘i (Fig. 1). The model was initially developed to identify such flooding in the City of Tybee Island, Georgia^26^. The method has been adapted as part of the present study to additionally account for contributions from the adjacent and contaminated Ala Wai Canal, an estuarine waterway that receives stormwater from the majority of storm water conduits in Waikīkī. Of particular concern is the potential for floodwaters originating from the Ala Wai Canal to enter heavily trafficked urban spaces within Waikīkī (Fig. 2), as waters are known to contain dangerous contaminants that include sewage^31^, microbiological pathogens^32^, and other urban contamination^33,34^. Many coastal cities similarly rely on estuarine waterways as receiving waters for gravity-fed stormwater networks, in which combinations of storm surge, tidal fluctuations, precipitation, and SLR can impede outflow, leading to prolonged urban flooding and backflow^35^. Understanding the complex interactions between coastal and fluvial influences on backwater conditions of stormwater infrastructure is crucial for predicting flood extents and developing effective flood management strategies.

Floodwaters additionally pose a public health concern due to precipitation-driven emergence of contaminated groundwater. Recent geochemical studies in Waikīkī indicate that aging sewage infrastructure contributes to groundwater contamination through sewage exfiltration, such that correlations have been made linking visitor arrivals with the detection of sewage tracer compounds^34^. Leaking sewage enters the groundwater and infiltrates cracked stormwater networks, further compounding the issue of contaminated flood waters^34,36^. As SLR lifts baseline groundwater elevations, groundwater emergence driven by both precipitation and tidal influence will likely increase. Such emergence by groundwater is an issue ubiquitous to low-lying urban areas^37,38^, emphasizing the need for consideration in infrastructure management.

### Study objective

The objective of this study was to develop a compound flood model capable of reproducing historical storm conditions and evaluating how flood characteristics evolve under projected SLR. A key aim was to identify critical SLR thresholds at which the dominant flood drivers shift, particularly where tidal influences begin to outweigh rainfall-runoff contributions. Additionally, the methodology facilitated the identification of flood sources known to contain substantial urban contamination.

Compound simulations were hydrologically calibrated to ensure reproduction of interactions between coastal and precipitation induced processes and validated using multiple lines of evidence to verify realistic representation of coastal, fluvial, pluvial, and subsurface processes. Each simulated storm was characterized by distinct spatial and temporal rainfall distributions, incorporating coastal forcing conditions from local tide gauge data, and precipitation forcing conditions from rain gauge corrected radar data. Simulations considering SLR utilize 0.3 m (1 ft) increments of SLR up to 1.2 m (4 ft), aligning with the National Oceanic Atmospheric Administration (NOAA) Intermediate scenario (RCP8.5), which projects approximately 0.3 m of SLR by 2050 and 1.2 m by 2100 for Honolulu, Hawai‘i^39^.

While the current study assumes stationarity for both dynamic forcing and static domain input, many recent studies suggest that climate-driven variables influencing compound flooding—such as precipitation, sea level, and storm-tide interactions—are increasingly non-stationary under climate change^40^. In Hawai‘i, however, climate projections remain highly uncertain due to inter-model disagreement, internal climate variability, and regional downscaling limitations^41-42^. As projection datasets continue to advance, future applications of this modeling framework should consider incorporating non-stationarity through bias-corrected climate projections, non-stationary frequency analysis, and dynamically evolving boundary conditions to more accurately represent long-term variability and flood risk.

The study employs the WRF-Hydro (version 5.2.0) model configuration in which the one-dimensional Noah-MP column land surface model^43,44^ is used to represent the moisture and energy fluxes between the atmosphere, land surface, and shallow subsurface. The Simple Water Balance model simulates hydrologic processes that include rainfall reaching the land surface and resulting infiltration into the soil column, as well as exfiltration and surface runoff^45^. Once the ponded surface water on a grid cell exceeds a specified threshold, overland flow is computed with an explicit, finite-difference, diffusive wave formulation using Steepest Descent methods^46,47^. Within fully saturated soil layers, lateral subsurface flow is determined using the quasi three-dimensional Boussinesq equation and steady-state approximation^48,49^. Urban drainage systems in Waikīkī are incorporated into the model using the U.S. Environmental Protection Agency (EPA) Storm Water Management Model (SWMM)^30^, wherein WRF-Hydro-CUFA the one-dimensional hydraulic solver is two-way coupled to the overland flow module of WRF-Hydro, allowing the exchange of floodwater information^26^. The coastal boundary component from WRF-Hydro-CUFA allows dynamic water levels to be forced across a user-specified coastline, accounting for tides, SLR, and other coastal inundation sources. Detailed development and validation of these model components are provided in their respective literature.

### Study area

Waikīkī is a densely urbanized coastal district and heavily patronized visitor destination. Its de facto population often exceeds 125,000 within a land area of 3.4 square kilometers, representing an effective population density comparable to that of Manhattan, New York. It is a vital economic hub, accounting for 7.3% of the state’s civilian jobs and 7.1% of all state tax revenue^50^. Once home to wetlands and fishponds, the area has undergone extensive land reclamation and development over the past century^51^. These transformations have resulted in a heavily paved and low-lying landscape, where approximately 78% of the land cover is impervious^,52^ and 97% of its ground surface lies within 3 m of local mean sea level (LMSL)^27^.

The Ala Wai Canal was constructed in the early 1920s to facilitate past development projects by draining natural floodplains and rerouting three streams that flowed across the Waikīkī area^51^. It delineates the northern boundary of Waikīkī, managing over 75% of the gravity-flow drainage from the Waikīkī area^29^. This is in addition to serving as the receiving waterbody for stormwater runoff from the 4,099-acre Mānoa-Pālolo watershed^53^. The canal is enclosed at its eastern end and opens to nearly 50 m wide at the Ala Wai Yacht Harbor, where it serves as a conduit to the Pacific Ocean, subject to both tidal and precipitation influences.

The canal is known to be heavily contaminated, receiving polluted discharge containing petroleum products, pesticides, fertilizers, and debris from upland streams, and discharging an estimated 23,350 acre-feet of runoff into the ocean annually^54^. The discharge is chronically host to fecal contamination and acts as receiving waters for occasional sewage spills. One major spill in 2006 released 48 million gallons of raw sewage into the waterway. This spill resulted in a hazardous public health situation, in which one person died from *vibrio vulnificus*-related sepsis following exposure to Ala Wai Canal waters^53^. *Vibrio*, a pathogen associated with brackish water and sometimes linked to sewage, has been found to have a chronic presence in the canal, in which moderate to prolonged rainfall events can produce favorable conditions for growth^55^*.* Despite ongoing mitigation efforts to reduce contamination, including emergency bypass systems and bioremediation projects, the canal remains a significant and largely unmanaged host of contamination, primarily due to persistent sewage leaks and stormwater runoff^56^.

### Local precipitation events

Rainfall patterns in Hawai‘i exhibit sharp spatial gradients, influenced by the complex island terrain and unique climate setting of the region^57^. Extreme rainfall events typically occur during the local wet season (November–April) and are often linked to four types of atmospheric disturbances: midlatitude fronts, Kona lows, upper-level disturbances, and tropical cyclones. These low pressure systems lift the trade wind inversion and enable deep convection, creating favorable moisture conditions for significant precipitation to occur^57-58^. A 20-year analysis investigating rainfall patterns over Oʻahu found that disturbances account for almost half of the total wet season rainfall, with Kona lows being particularly contributory, making them a key factor in determining whether a season is particularly wet or dry^59^. These findings corroborate previous research establishing Kona storms as a significant contributor to wet season rainfall^58,60^. Future projections suggest the annual average rainfall in Hawai‘i is expected to increase by approximately 8%, with extreme rainfall rates projected to rise by over 20% during the dry season and by about 10% during the wet season^61^. These changes are attributed to complex orographic interactions, resulting in varied precipitation patterns. Therefore, it is important to consider multiple storm types with varying spatial and temporal characteristics when preparing for flood risks in Hawai‘i.

For this study, three recent heavy rainfall events that caused flooding within the modeling domain were simulated for use in building and validating the flood model. Two of these events were associated with Kona lows, while the third resulted from an upper-level disturbance. The first event, a 50-year Kona storm in December 2021 (‘KS-2021’), caused extensive flooding in parts of Waikīkī. The second, a 5-year upper-level disturbance in April 2023 (‘ULD-2023’), led to localized flooding within the Ala Wai Watershed, though impacts in Waikīkī were minimal. The final event, a late-season 5-year Kona storm in May 2024 (‘KS-2024’), led to widespread flooding in Waikīkī and concern regarding the effectiveness of the area’s drainage systems^62^. By analyzing storms with varying rainfall distributions, meteorological triggers, and coinciding tidal conditions, this study aims to capture a range of hydrological responses, increasing the robustness of its findings. For more information on the storm events used in this study, refer to Supplementary Information.

## Results

The initial stage of this study included development and validation of the compound flood model. This was done by constructing and calibrating a model using data from the KS-2021 storm event, then verifying its ability to reproduce observed flooding and Ala Wai Canal tailwater conditions during the ULD-2023 and KS-2024 storm events. Calibration and validation are presented in three parts: 1) calibration and validation using measured canal water levels, 2) validation of inland flooding using photographic documentation, and 3) validation of subsurface hydrologic behavior using measured groundwater levels. Following validation, SLR forcing was applied to the KS-2021 and KS-2024 storms. The ULD-2023 storm was included in the initial stages to validate canal water levels and subsurface moisture conditions, based on the availability of observations, and to confirm the absence of surface flooding in the present-day simulation. However, because the event did not produce notable flooding in Waikīkī, it was excluded from the future projected SLR flood simulations.

Flood simulations described here represent maximum floodwater depths and extents produced during the storm events described above. The maximum surface water head relative to the ground surface was used to represent floodwater depth. Water depths less than 0.05 m were excluded from the analysis to avoid considering thin veneers of water that commonly appear across the model grid during heavy rainfall events, but do not represent meaningful surface flooding. The following sections present the results of model calibration and validation, along with assessments of the potential for increased backflow and the emergence of contaminated waters under SLR scenarios, applied in 0.3 m increments, up to 1.2 m, to the KS-2021 and KS-2024 storm events.

### Model calibration and validation of compound flood sources

Model calibration was conducted to ensure that simulations captured processes generated by rainfall-runoff within the Ala Wai watershed, in conjunction with tidal influence; thus, ensuring that canal waters remained influenced by both land- and sea-based processes. Because the occurrence of the KS-2021 event motivated the present study, Ala Wai Canal tailwater observations were not yet being collected. However, data collected at a U.S. Geological Survey (USGS) water level gauge located upstream of the Ala Wai Canal in the Mānoa-Pālolo Drainage Canal (MPDC, see Fig. 1 for location), just outside of the main area of interest for this study, displayed both tidal and precipitation signals. Modeled surface water head at the grid cell location representative of the MPDC water level gauge was used to ensure that both the tidal signal and precipitation-induced pulses were simultaneously observed with the appropriate timing, in the simulated time series for each of the three storms (see Supplementary Fig. 1). The analysis illustrates the model’s ability to capture compound flood processes across all three storms, emphasizing the importance in accounting for both precipitation and coastal water levels in complex coastal settings. In addition, model calibration included sensitivity testing of the model time step, as well as optimization of spatial resolution to ensure tidal signals and water flow through waterways were not unrealistically restricted.

**Fig. 3:**
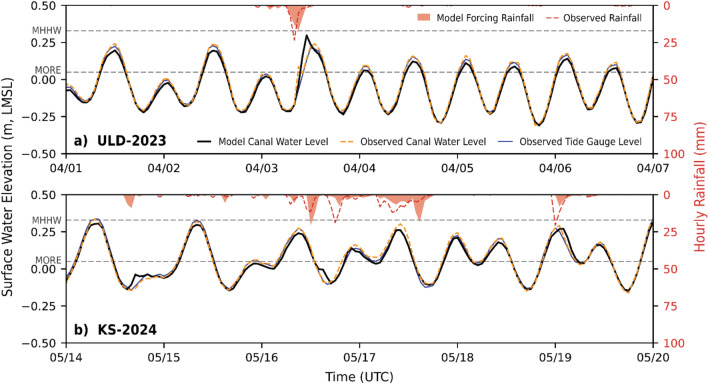
Simulated and observed canal water levels and rainfall during two storm events for which Ala Wai Canal tailwater conditions were recorded: **(a)** ULD-2023 and **(b)** KS-2024. Shown in the upper sections of each panel are hourly rainfall observations (dashed red) and model forcing (red shaded) from the Pālolo Fire Station gauge. Shown in the lower sections of each panel are the canal water level simulations (solid black) and observations (dashed orange) captured at the site of the Ala Wai Canal transducer. Also shown are hourly observations from the Honolulu tide gauge (solid blue), used to force water levels at the mouth of the Ala Wai Canal and along the coastline. For reference, the Mean Higher High Water (MHHW) tidal datum and Mean Outfall Rim Elevation (MORE) are indicated as dashed black horizontal lines.

Model validation using measured water levels was performed by using the previously calibrated model from the KS-2021 storm to compare the time series of simulated and observed tailwater elevations in the Ala Wai Canal at the transducer location for the ULD-2023 and KS-2024 storm events (Fig. 3). Observed canal water level data was smoothed using a 30-min rolling average and resampled to 1-h intervals to match the temporal resolution of the model output. Simulated and observed Ala Wai Canal water levels illustrate the ability of the model to reproduce influence from tides and precipitation on water levels within the canal. These comparisons reflect the combined influence of rainfall-runoff and tidal effects, demonstrating the model’s ability to capture appropriate magnitudes of this interaction.

Model skill was evaluated using several statistical measures comparing simulated and observed water levels in the Ala Wai Canal, including the Pearson’s correlation coefficient (*r*), the coefficient of determination (*R*^*2*^), the Nash–Sutcliffe Efficiency (*NSE*), and the Root Mean Square Error (*RMSE*). Simulated water levels showed strong agreement with observations, yielding an *r* = *0.935* and *R*^*2*^ = *0.874* for ULD-2023, and *r* = *0.944* and *R*^*2*^ = *0.891* for KS-2024. Model performance was further supported by *NSE* values of *0.865* and *0.883*, and *RMSE* values of *0.0525* m and *0.0458* m, for ULD-2023 and KS-2024, respectively. The simulated tidal signal closely followed the magnitude and timing of verified observations from the Honolulu tide gauge, which was used to force the model along the coast and at the mouth of the Ala Wai Canal. Instances where canal water levels deviated from tide gauge measurements corresponded to observed peaks in rainfall, highlighting the simulated interaction between tidal and precipitation forcing.

While minor discrepancies exist between observed rainfall and the model-forced rainfall at the corresponding location, the timing and magnitude of precipitation were generally well captured across storm events. Figure 3 shows observed and model-forced rainfall at the nearest rain gauge located at the Pālolo Fire Station for reference. By examining storms with varying combinations of rainfall intensity and tidal stage, these results demonstrate that the timing and co-occurrence of these drivers play a critical role in shaping compound flood severity.

### Validation of inland flooding using photographic documentation

**Fig. 4:**
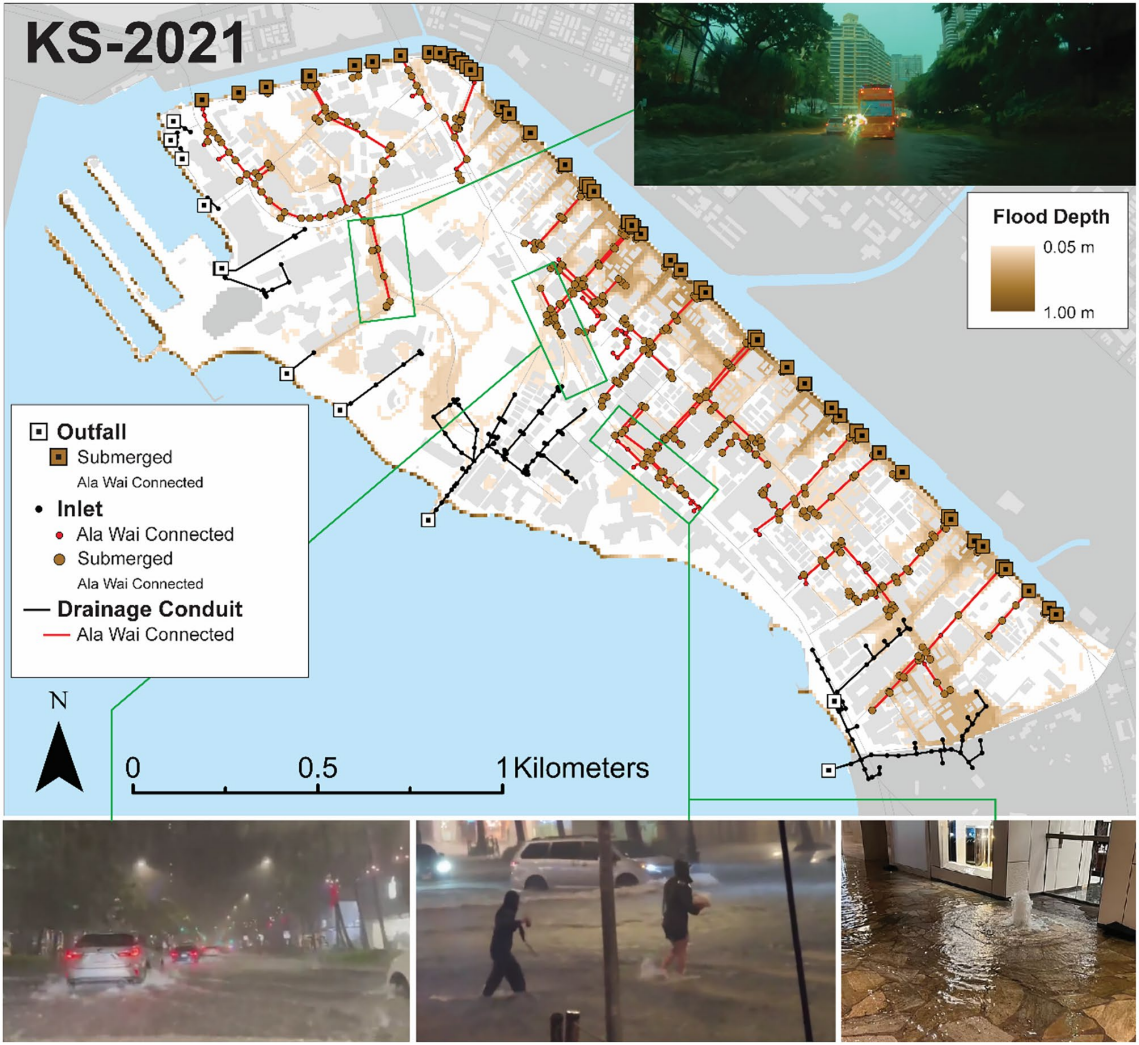
Comparison of maximum simulated surface head with flood imagery from the KS-2021 storm event. Locations of potential drainage failure were identified, where images of geysering at drainage inlets in central Waikīkī support the likelihood of backflow directly from the Ala Wai Canal. The imagery also documents public exposure to floodwaters, as well as water depths sufficient to generate wakes from passing vehicles. Top Right: Photograph by Hawaii John; Bottom Left: Photograph by Michael Helms; Bottom Center: Photograph by Paul Roszkowski; Bottom Right: Photograph by Natasha Tsuji.

**Fig. 5:**
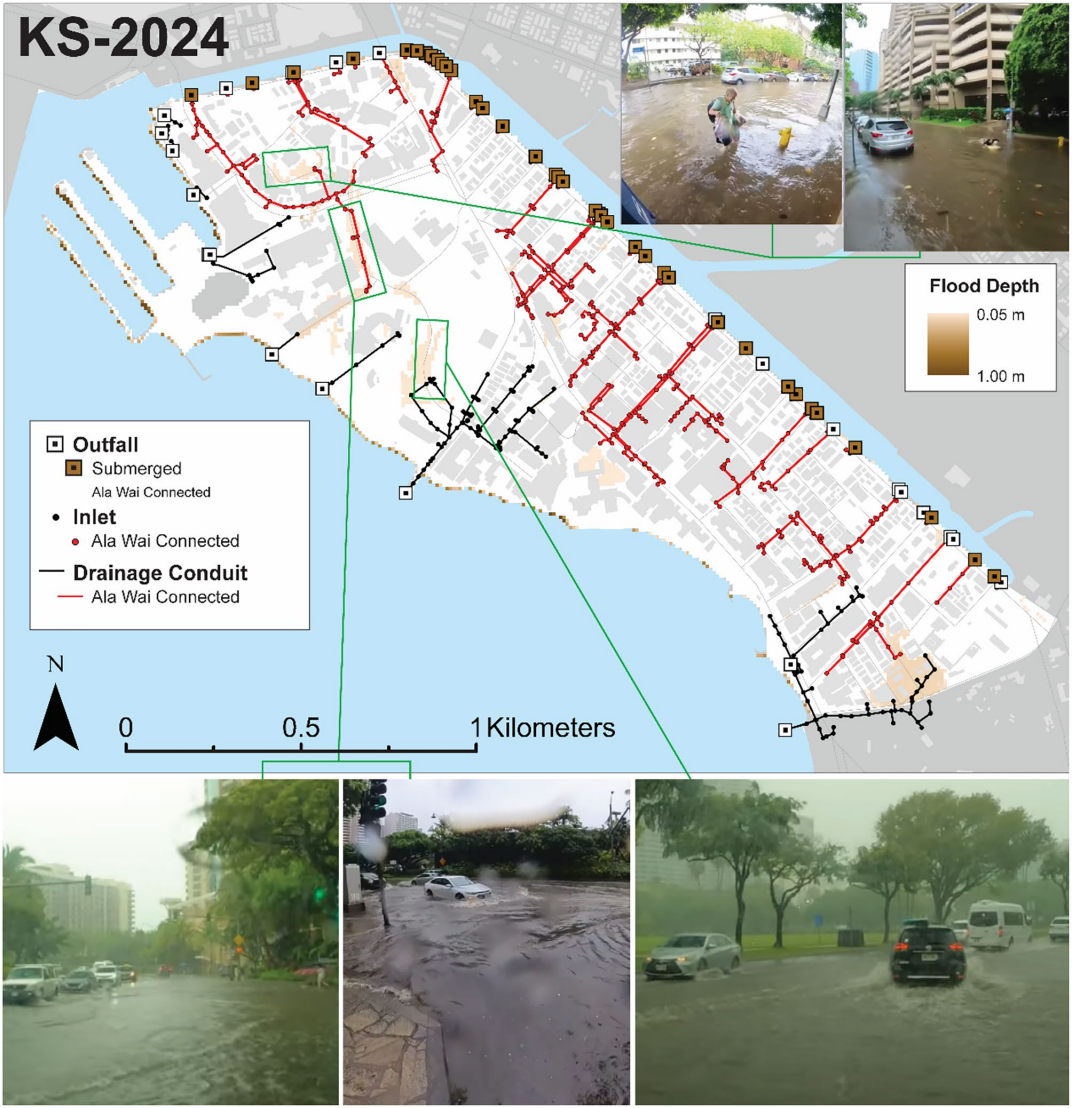
Comparison of maximum simulated surface head with flood imagery from the KS-2024 storm event. Locations of potential drainage failure were identified, revealing that backflow likely did not occur during this event. However, nearly all drainage outfalls were exceeded, suggesting that flooding was exacerbated by blocked outfalls. This is particularly true of a northwestern location in Waikīkī, which media reports highlighted as experiencing drainage issues during the event^62^. Top Right: Photographs by David Muther; Bottom Left and Bottom Right: Photographs by Hawaii John; Bottom Center: Photograph by David Muther.

Model validation using flood imagery was qualitatively accomplished following a methodology similar to that used in the original development of WRF-Hydro-CUFA^26^. Imagery captured during respective storm events were sourced from media outlets and compared to simulations of maximum flooding representing the KS-2021 (Fig. 4) and KS-2024 (Fig. 5) storm events. To quantify the impact of urban drainage systems on flooding, simulated maximum head elevations along the Ala Wai Canal were used to identify two indicators of drainage failure, following the methodologies of recent studies^28,29^: (1) flow blockage at outfalls and (2) reverse flow from the canal to the ground surface. The potential for blocked drainage is assessed by identifying outfall locations along the drainage canal, where rim elevations were exceeded by simulated maximum tailwater levels. Similarly, to evaluate the potential for drainage backflow, inlet locations connected to the Ala Wai Canal through conduits were identified where inlet elevations were exceeded by simulated maximum tailwater conditions.

While photographic evidence of flooding in Waikīkī was available for the KS-2021 and KS-2024 storm events, there was no evidence indicating significant surface flooding within the study area during the ULD-2023 event. Observations of drainage system water levels reported in a previous study^29^ that captured the ULD-2023 event, suggest that the Waikīkī drainage system was not over capacity. The simulation produced as part of this study aligned with this finding, validating the model’s ability to reproduce the absence of flooding during a minor event.

Overall, the model accurately reproduces flooding at observed locations, with simulated flood depths comparable to those indicated by vehicles traversing through floodwaters. Results reveal widespread drainage inefficiencies throughout much of Waikīkī, primarily due to submerged outfalls, as shown in both the KS-2021 and KS-2024 simulations. The KS-2021 simulation suggests that 400 of the 444 Ala Wai Canal-connected drainage inlets were exceeded. During this event, multiple drainage inlets near the heavily trafficked Royal Hawaiian Shopping Center were observed geysering (Fig. 4), consistent with locations identified as backflowing in the simulation. In the surrounding area, the model produced flood depths between 0.1 and 0.2 m. Another example shown in Fig. 4 illustrates sidewalk overtopping in areas where the model simulated flood depths up to 0.15 m, consistent with photographic evidence of pedestrians crossing a street through ankle-deep water. Simulated drainage failures were also evident in the absence of rainfall. When rainfall was excluded from simulations, most outfalls (44 out of 51) remained submerged due to tidally driven tailwater levels in the Ala Wai Canal. These findings indicate that rainfall-induced flooding in this district is intensified by reduced drainage capacity and tidal backwater effects that limit outfall performance.

During the KS-2024 simulation (Fig. 5), tidal and precipitation forcing were relatively mild, and no backflow was identified at any of the drainage inlets. However, simulations indicate an exceedance of 36 of the 51 outfalls, including one in northwestern Waikīkī responsible for draining areas that frequently flood, as documented in imagery from both events. This suggests that precipitation-induced flooding in these areas is exacerbated by outfall blockage, caused by impedance of drainageways and reduced capacity due to inundation from Ala Wai Canal waters. The simulation results indicate that while outfalls were submerged below Ala Wai Canal tailwater levels, inlets remained above tailwater levels, meaning conditions for direct backflow had not been reached. However, news reports documented drainage failures specifically in northwestern Waikīkī, aligning with simulations that show flood depths and submerged outfalls connected to drainage inlets, which would have impeded functional drainage.

### Comparisons of soil moisture conditions to groundwater levels

Tidally corrected groundwater level observations were used to assess the model’s ability to simulate shallow subsurface flow behavior during storm events. Simulated volumetric soil moisture for the 2-m soil column served as a proxy for evaluating these conditions. The model computes volumetric soil moisture for each of the four user-defined soil layers, and a depth-weighted average was computed from these values to represent overall soil moisture across the full soil column. Hereinafter, references to modeled soil moisture refer to the depth-weighted average.

**Fig. 6:**
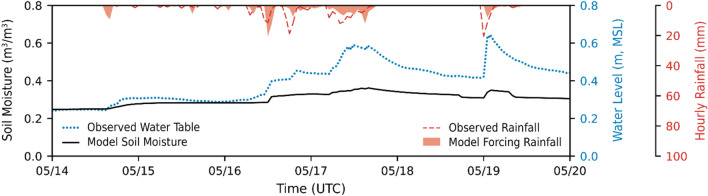
Time-varying soil moisture conditions during the KS-2024 storm event at a location representative of active groundwater monitoring in central Waikīkī. Simulations of soil moisture (solid black) are superimposed upon tidally corrected observations of groundwater elevations (dotted blue) recorded during the subject storm, along with both observed (dashed red) and model-forced (red shaded) hourly rainfall for the Pālolo Fire Station monitoring location, illustrating the model’s ability to simulate realistic changes in saturated subsurface conditions generated by precipitation-induced forcing.

Within the Waikīkī domain, groundwater levels were recorded at a centrally located groundwater monitoring well during the KS-2024 event (Fig. 6). The groundwater levels were used to verify appropriate changes in soil moisture simulated by the model during conditions of rainfall. At the monitoring site, variations in simulated soil moisture correspond with the observed rise in groundwater levels. The comparison of observed groundwater levels with modeled soil moisture demonstrated that the model approximately captures saturated subsurface behavior, a key factor influencing the timing and magnitude of surface head ponding and overland flow in the model.

### Future projected flooding considering sea-level rise

To better understand the progression of flooding under SLR, the KS-2021 and KS-2024 storm events were simulated considering 0.3 m increments of SLR, up to 1.2 m. The KS-2021 and KS-2024 events represent extreme and mild conditions, respectively, in terms of both rainfall and tidal magnitude. The KS-2024 event represents a tidal elevation lower than that of the average daily high tide, such that its simulation represents one of daily exceedance, while the KS-2021 event is more representative of a biennially occurring extreme tide event^63^. To quantify the contributions of precipitation verses tidally driven flooding, simulations were conducted both with and without rainfall. The role of Ala Wai Canal tailwater conditions in driving drainage inefficiencies and backflow was again assessed by identifying submerged outfalls and backflowing inlets considering both events, with and without rainfall, and under incremental SLR.

**Fig. 7:**
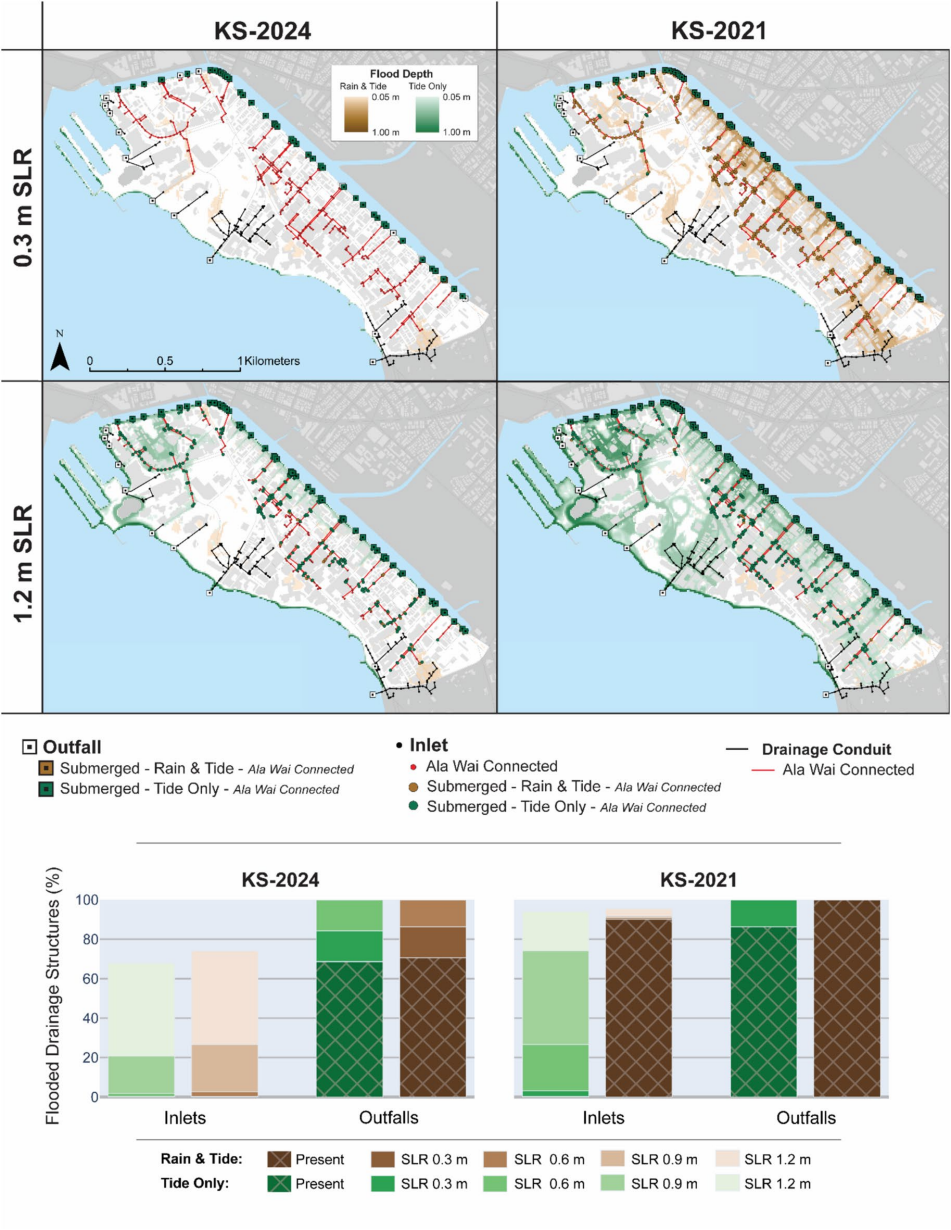
**Top**: Maximum simulated flood depths and extents in Waikīkī considering the KS-2024 and KS-2021 storm events under 0.3 m and 1.2 m SLR. These simulations illustrate flooding generated by precipitation-driven events (brown gradient) and blue-sky conditions (green gradient), with the latter superimposed on the former. Brown squares indicate locations where simulated tailwaters exceed Ala Wai Canal-connected outfalls, signifying decreased drainage efficiency; green squares indicate submerged outfalls under tidal forcing alone; brown circles identify locations where tailwaters exceed Ala Wai Canal-connected drainage inlets, highlighting potential pathways for backflow; and green circles indicate submerged inlets under tidal forcing alone. **Bottom**: Percentage of flooded inlets and outfalls within the Waikīkī study area across 0.3 m increments SLR, up to 1.2 m, for KS-2024 and KS-2021, both with precipitation (brown gradient) and without precipitation (green gradient).

Figure 7 maps simulated flood depths for the KS-2021 and KS-2024 events under the 0.3 m and 1.2 m SLR scenarios, both with rainfall (“Rain & Tide”) and without rainfall (“Tide Only”). It also summarizes flood depths across all SLR scenarios evaluated in this study, including present-day conditions and SLR increments of 0.3 m, 0.6 m, 0.9 m, and 1.2 m, under both rainfall forcing conditions. Simulations indicate that even in the absence of rainfall, the combination of tides and SLR will generate extensive flooding, largely from a heavily contaminated source.

In the KS-2024 event, reverse flow becomes an issue at a small number of locations with 0.6 m SLR, while SLR of 0.9 and 1.2 m results in widespread backflow, affecting more than 100 and 400 Ala Wai Canal-connected inlets, respectively, regardless of precipitation. In contrast, during the more intense KS-2021 event, nearly all outfalls are exceeded under both sunny-day and rainfall conditions at present-day sea level, and reverse flow from Ala Wai-connected inlets becomes an issue at a small number of locations with just 0.3 m SLR. As sea levels rise, the number of backflowing inlets increases dramatically, with more than 100 inlets affected at 0.6 m SLR and most inlets backflowing at 1.2 m SLR. Although canal water level data were unavailable for validation, simulations suggest that nearly all inlets likely became sources of backflow during the KS-2021 event under present-day conditions (Fig. 4). Overall, simulations considering both events suggest that under blue-sky conditions (i.e., no rainfall), backflow from the Ala Wai Canal may begin with as little as 0.3 m SLR during extreme tidal conditions (KS-2021), and with 0.6 m SLR under more moderate daily tidal conditions (KS-2024).

**Fig. 8:**
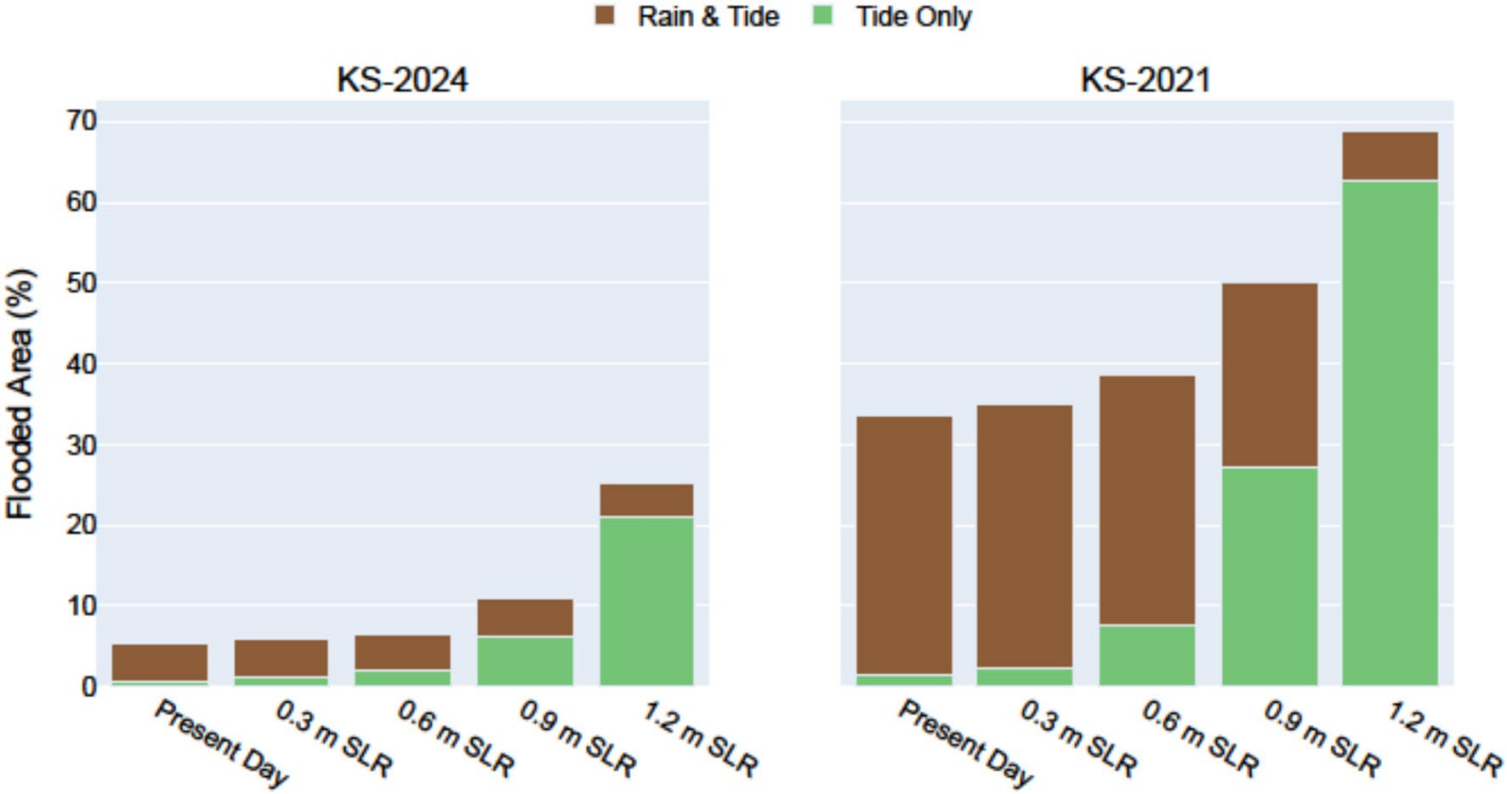
Percentage of area flooded in Waikīkī considering KS-2024 and KS-2021 storm events, under 0.3 m increments of SLR, up to 1.2 m. Simulations include scenarios of precipitation-driven events (brown) and blue-sky conditions (green), to better understand flooding contributions from each source. As sea levels rise, tidal contributions become the primary driver of flooding.

With respect to the total land area flooded, the relative contribution of rainfall to overall flooding diminishes progressively with SLR for both storm events (Fig. 8). With 0.3 m SLR, precipitation forcing during the KS-2024 event accounts for 78% of the total flooded area, dropping to only 17% with 1.2 m SLR, at which point more than 25% of the Waikīkī area is projected to be inundated. Similarly, in the more extreme KS-2021 event, precipitation contributes to 93% of the total flooded area at 0.3 m SLR, but only 9% at 1.2 m SLR, where more than 68% of the Waikīkī study area is projected to be inundated. These findings suggest that while rainfall events can exacerbate SLR-induced flooding, the dominant sources of inundation will increasingly be those which occur more chronically. Furthermore, because much of the flooding is directly connected to the Ala Wai Canal via storm drainage infrastructure or overbank flow, floodwaters will originate not from a clean marine source, but from a waterway heavily contaminated by runoff from nearly an entire urban watershed.

### Future projected changes in soil moisture considering sea-level rise

**Fig. 9:**
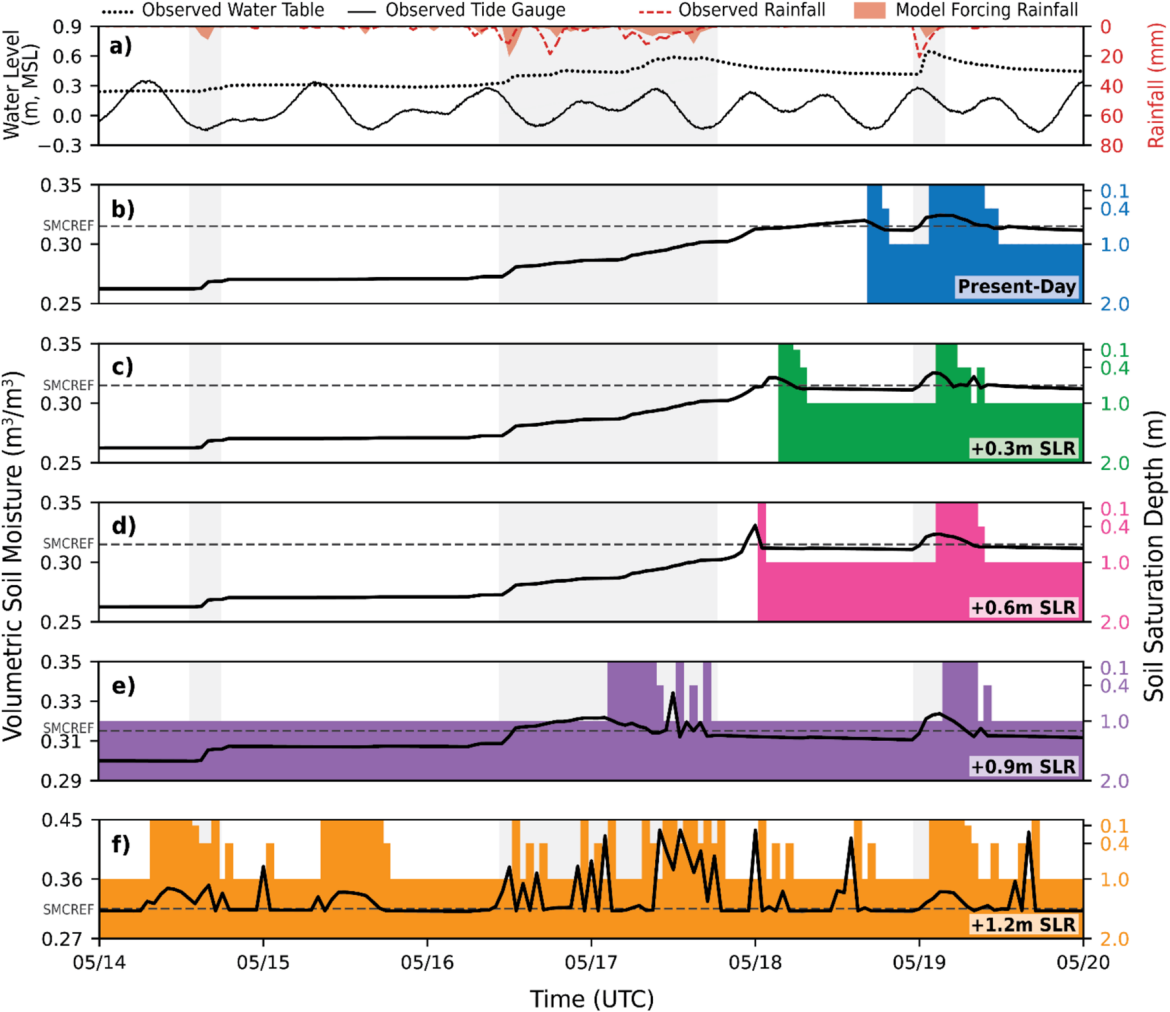
Simulated and observed subsurface hydrologic conditions at a groundwater monitoring site in central Waikīkī, along with observed forcings from nearby locations, during the KS-2024 storm event. **(a)** Observed tidally corrected groundwater levels (dotted black) at the monitoring well, along with forcings that influence subsurface moisture conditions: observed water levels from the Honolulu tide gauge (solid black) and hourly rainfall from the Pālolo Fire Station, including both observed (dashed red) and model-forced (red shaded) rainfall. Bottom panels show simulated volumetric soil moisture (solid black) and soil saturation depth (colored shading) for five SLR scenarios: **(b)** present-day, **(c)** 0.3 m, **(d)** 0.6 m, **(e)** 0.9 m, and **(f)** 1.2 m. The dashed horizontal line in each panel marks the reference soil moisture content (SMCREF) at the evaluated location. As SLR increases, the soil column remains closer to saturation, limiting infiltration capacity and increasing the incidence of groundwater emergence, which eventually occurs in the absence of rainfall under 1.2 m SLR. Note: The left y-axis range for volumetric soil moisture is adjusted in panels (e) and (f) to better capture variations. The right y-axis for soil saturation depth uses tick marks aligned with the depths of the four model soil layer depths.

Simulated changes in soil moisture and soil saturation depth under incremental SLR of 0.3 m, up to 1.2 m, are shown for the location of a central Waikīkī monitoring well, where groundwater levels were recorded during the KS-2024 event (Fig. 9). Simulated soil saturation depth is derived from model output of water table depth, which is defined as the vertical distance from the land surface to the top of the saturated soil layer nearest the surface. To visualize this as a bar plot representing the thickness of the saturated zone, simulated water table depth is inverted so that the bars extend upward from the bottom of the soil column located at a depth of 2 m to the land surface at 0 m.

In the model, water table depth is governed not only by total soil moisture content, but also by the vertical distribution of moisture within the soil column and the resulting pressure head. The reference soil moisture (SMCREF), also known as field capacity, represents the volumetric soil moisture threshold above which gravity drainage begins. At the evaluated location, SMCREF is 0.315. Below this threshold, moisture is retained in a soil layer by capillary forces; above it, excess moisture can drain downward. Across all simulated scenarios, the water table did not rise to the surface until soil moisture in all layers exceeded SMCREF. This suggests that although rainfall may rapidly saturate the uppermost soil layers, the rise of the water table, and thus the increase in soil saturation depth, is largely dependent on whether deeper soil layers reach SMCREF.

Under present-day conditions, modeled soil moisture responds to rainfall in a manner consistent with observed groundwater level fluctuations. With 0.3 m SLR, soil moisture follows a similar trend but reaches saturation more rapidly, as slightly elevated antecedent moisture reduces infiltration capacity. At 0.6 m SLR, infiltration capacity diminishes even earlier, though transient drainage or lateral subsurface flow allows for additional infiltration following fully saturated soil conditions. At 0.9 m SLR, both soil moisture and saturation depth are elevated even before the first rainfall episode, significantly reducing infiltration capacity, which ceases entirely as the second rainfall episode intensifies. Under 1.2 m SLR, soil moisture fluctuations at intervals indicative of tidal influence suggest that infiltration no longer occurs, leading to ponding as tidal waters inundate low-lying areas with no drainage outlet. Groundwater emergence also becomes increasingly evident, occurring even prior to the onset of rainfall.

## Discussion

Studies on SLR-related flood impacts often focus primarily on surface damage and economic losses, while frequently overlooking contamination risks from floodwaters. This is despite well-documented evidence that urban runoff from heavy rainfall can carry contaminants, posing public health risks through direct exposure^64^. As SLR progresses, flooding will increasingly stem from compound sources, introducing contaminated floodwaters through multiple pathways, complicating management efforts. This study highlights two key sources of contamination that will become more prevalent with SLR: (1) storm drain failure, which will increasingly transport heavily contaminated waters from an adjacent urban waterway through drainage conduits and into heavily trafficked urban areas; and (2) groundwater emergence, which actively receives sewage and other contaminants from aging and deteriorating sewage infrastructure.

The Ala Wai Canal is designated as an impaired waterbody under the Clean Water Act as the result of hosting high levels of contaminants, including nitrogen, phosphorus, pathogens, and pesticides^65^. Numerous studies have confirmed the chronic presence of sewage-related pathogens in canal waters^31,66-67^. During rainfall events, pathogen concentrations rise in the Ala Wai Canal, caused in part by resuspension of contaminated sediments and altered salinity conditions that can promote microbial growth, frequently exceeding health agency thresholds^31^. Such increases in contamination coincide with flood risks, especially in the low-lying, highly trafficked Waikīkī, where this study identifies conditions conducive to backflow and the spread of contaminated waters under rising sea levels.

The associated public health risks are well-documented, particularly among residents and users of the Ala Wai Canal, who have reported infections caused by Methicillin-resistant *Staphylococcus aureus* (MRSA)^68^. *Staphylococcus aureus*, a bacterial pathogen linked to septicemia, pneumonia, and skin infections^69^, is the leading cause of bacterial fatalities worldwide^70^. First identified in hospitals in 1960, MRSA has become increasingly prevalent in community settings since the 1990s^69–72^. In Hawai‘i, MRSA infections contribute to as many as 200 deaths annually, potentially twice the observed rate in other regions^73^. This highlights the critical need to address sources of contaminated flooding in high-exposure areas like Waikīkī.

Tidally influenced drainage systems may also serve as reservoirs for MRSA and other sewage-related pathogens. Studies suggest that saline, turbid environments with limited sunlight promote the survival of *Staphylococcus aureus*^70^. Another pathogen of concern in Hawai‘i waters is *Vibrio*, a bacterium capable of causing necrotizing fasciitis. *Vibrio* has been detected in the Ala Wai Canal, with higher prevalence observed following modest rainfall^55^. Notably, exposure to *Vibrio* in the Ala Wai Harbor has been linked to a fatal infection following a rain event and resulting multi-million-gallon sewage spill^53^. Recent research indicates that storm drains in low-lying areas increasingly act as pathways for untreated wastewater, in which sewage contamination exfiltrates from deteriorated sewer lines into coastal groundwater, which then infiltrates into similarly deteriorated drainage conduits^36^. As sea level continues to rise, elevated tailwater conditions in the Ala Wai Canal will increasingly cause these contaminated waters to surface, initially during rainfall and eventually during daily high tides.

In addition to surface flooding, groundwater emergence represents an additional pathway for sewage contaminated waters to enter public spaces. A recent study projects that with 1 m of SLR, approximately 23% of Waikīkī will experience groundwater emergence, not considering the influence of rainfall^74^. The same study found that 86% of active cesspools in the area currently contribute to contamination of shallow groundwater. Local geochemical analyses further confirm sewage contamination from aging conduit systems, with sewage tracers correlating with daily visitor arrivals. These findings reinforce a call by the American Society of Civil Engineers (ASCE) for continued maintenance and replacement of aging sewage and stormwater infrastructure. A recent evaluation gave Hawai‘i’s critical infrastructure a D+ grade, citing challenges such as aging infrastructure, insufficient funding, population growth, and SLR^79^. Strengthening wastewater and storm drain infrastructure is therefore essential to mitigate chronic exposure risks and enhance long-term community resilience.

Persistent shallow groundwater and reduced soil infiltration under rising sea levels further compound these challenges by undermining infrastructure resilience in low-lying urban environments. As the soil column remains near saturation, infiltration capacity decreases and shallow groundwater persists for longer durations, leading to chronic subsurface saturation. These conditions weaken building foundations, promote corrosion of buried utilities, and reduce the structural integrity of roads and drainage conduits, causing long-term degradation, increased maintenance costs, and heightened safety risks^37,38^.

Beyond physical and public health impacts, rising contaminated groundwater in Waikīkī also carries serious cultural implications. Waikīkī is revered as a place with deep historical and cultural significance, containing subsurface cultural resources such as iwi kūpuna (ancestral remains), remnants of ancient habitation, and sites tied to its role as a royal retreat. The interaction between contaminated groundwater and these culturally sensitive sites raises concerns regarding the responsibility to preserve and protect these heritage resources. Safeguarding these sites is essential for maintaining cultural integrity and honoring their historical significance.

As SLR becomes the dominant driver of flooding via direct marine inundation, groundwater emergence, and storm drain backflow, rainfall will play a progressively smaller role in overall inundation dynamics. This shift underscores the need for adaptive flood management strategies that explicitly account for compound flood conditions driven by tidal and groundwater processes. Ongoing studies in Waikīkī, informed by local SLR flood modeling efforts, are exploring innovative management approaches to mitigate these evolving hazards^75-76^. Incorporating floodwater contamination and water quality considerations into these efforts will strengthen risk assessments and inform targeted strategies to address contamination and protect public health. These findings also have implications for other low-lying coastal communities hosting shallow groundwater or that rely on contaminated estuarine waterways for stormwater management.

## Summary and conclusion

This study employs the advanced WRF-Hydro-CUFA flood model to simulate the multifaceted drivers of flooding, including pluvial, fluvial, coastal, storm drain-driven, and subsurface flood dynamics produced by recent precipitation events. It assesses compound and escalating SLR-induced flood threats affecting Waikīkī, Hawai‘i, a low-lying, densely developed, and heavily trafficked district with multiple pathways for flood exposure. While storm-generated runoff is known to carry contaminants hazardous to human health, Waikīkī faces additional contamination risks from groundwater emergence and backflow from an adjacent estuarine drainage canal.

Results of this study rely on reproductions of compound flooding generated by recent storm events, as well as the application of incremental SLR to modeled environmental forcing. Additional simulations that consider only tidal and SLR forcing were performed to distinguish coastal verses precipitation-driven flooding contributions. For each scenario, model results identify flooded areas and highlight potential sources of contamination due to storm drain dysfunction.

The results indicate that impaired drainage can be impactful under present-day conditions, even during moderate tidal and rainfall events. During more extreme tidal and rainfall conditions, widespread stormwater backflow is shown to originate directly from a heavily contaminated source as tailwater conditions become elevated. With SLR, widespread backflow is projected to occur even in the absence of rainfall, beginning at 0.9 m SLR considering mild tidal conditions, and 0.6 m SLR under extreme tidal conditions considered in this study. This suggests that waters contaminated with sewage-related pathogens will become a more persistent and chronic presence in the area.

At present, there are no highly effective management efforts being undertaken to curb complex contamination issues for this waterway or coastal groundwater. The results underscore the significant role that tidally influenced drainage conduits play in flooding and as a potential, progressively increasing source of dangerous contamination into heavily trafficked public spaces. This highlights the need to reevaluate existing drainage infrastructure, as well as the role of contaminated waters in future flooding, and to incorporate such considerations into flood risk management strategies to wholistically address complex challenges posed by systemic urban contamination.

While this study focused on reproducing recent observed flood events, the WRF-Hydro-CUFA framework offers a transferable approach for evaluating interactions among rainfall, tides, drainage, and groundwater in other low-lying coastal cities. Future applications could be improved by incorporating statistically derived design storms and accounting for non-stationarity to identify recurrent flood hotspots, exceedance thresholds, and contamination exposure zones. Establishing long-term monitoring networks in key waterways and storm drain systems will improve data availability for model calibration and validation, enhancing predictive accuracy and supporting the development of sustainable infrastructure and public health strategies. The findings highlight the urgent need to modernize stormwater and wastewater infrastructure, strengthen maintenance and monitoring programs, and integrate contamination risk into coastal flood mitigation planning. Enhancing public health preparedness through early warning systems and response protocols will be essential to improving urban resilience against evolving compound flood hazards.

## Methods

**Fig. 10:**
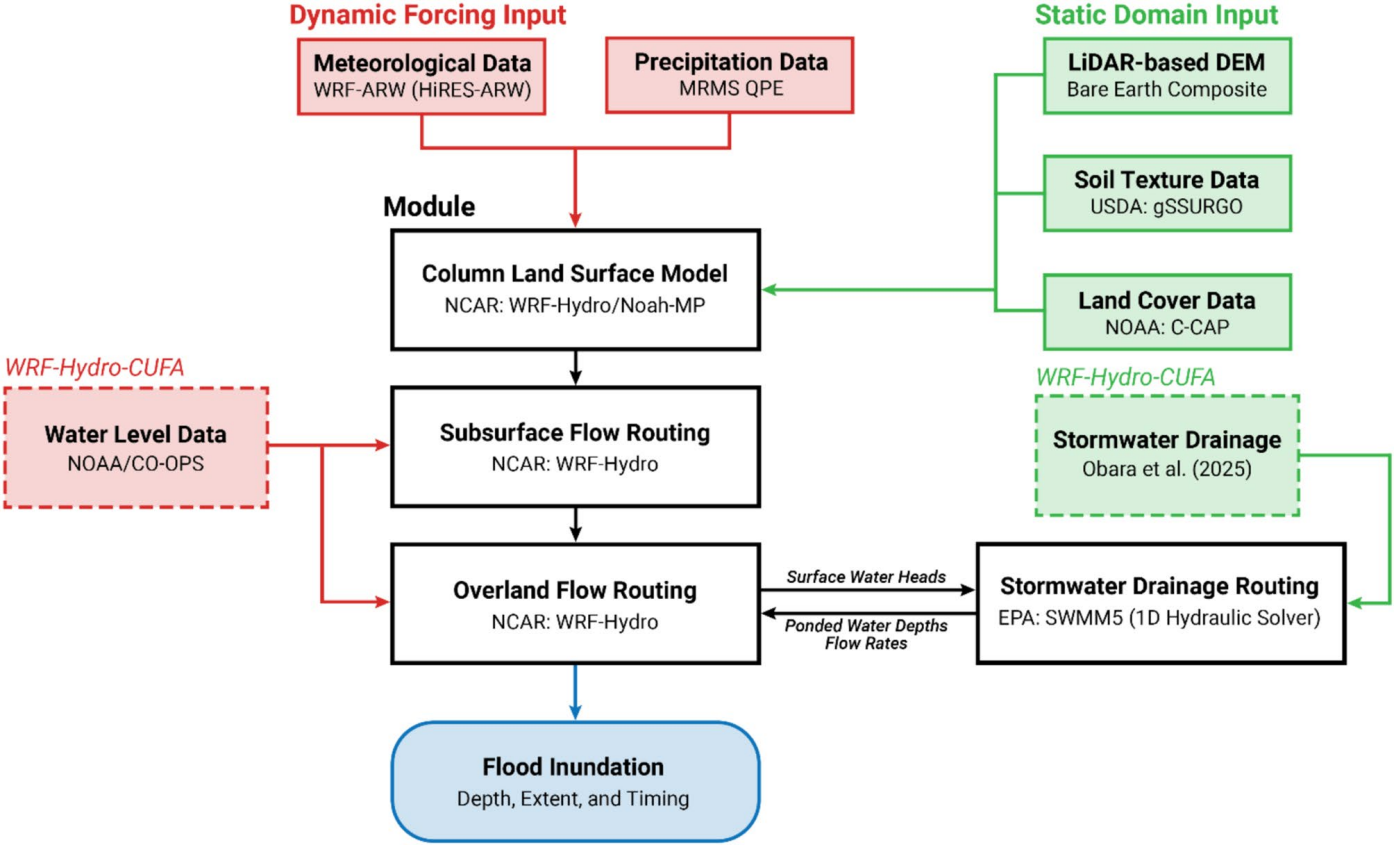
Schematic flow diagram of the WRF-Hydro-CUFA model configuration, illustrating key inputs, processes, and outputs. Dashed boxes denote inputs specific to the WRF-Hydro-CUFA implementation. Adapted from Son et al. (2023).

WRF-Hydro-CUFA was used to simulate compound flood inundation in Waikīkī, Hawai‘i. The modeling framework integrates the WRF-Hydro model with additional components developed for coastal-urban flood applications. Together, the model captures the combined effects of rainfall-runoff, coastal forcing, and urban drainage on flood dynamics to simulate flood inundation depth, extent, and timing. The model requires static domain inputs to define physical and geographic characteristics, and dynamic forcing inputs to represent time-varying meteorological and coastal water level conditions. Additionally, observational datasets are used for model calibration and validation. A schematic flow diagram of WRF-Hydro-CUFA inputs, outputs, and model components is provided in Fig. 10.

### Model configuration

WRF-Hydro (version 5.2.0) is a multi-scale, fully distributed modeling system that simulates the movement of water through the hydrologic cycle and serves as the foundation for the U.S. National Water Model (NWM) (version 2.1). The model couples the one-dimensional Noah-MP^43,44^ land surface model (LSM) with the terrain routing modules of WRF-Hydro. Surface runoff and infiltration are computed using the Simple Water Balance model, while lateral subsurface flow within saturated layers is represented by the quasi-three-dimensional Boussinesq equation under a steady-state approximation^48,49^. When surface head exceeds the maximum retention depth of a grid cell, overland flow is solved using an explicit, fully unsteady, finite-difference, diffusive wave formulation based on Steepest Descent methods^46,47^.

In WRF-Hydro, channel routing is implemented as a one-way coupling with the overland flow routing module, meaning that while water can enter channel grid cells, overbank flow does not feed back into overland flow processes. Therefore, we run the model with channel routing turned off, and simulate surface flow as an explicit overland process, mirroring previous approaches^12,26^. A detailed description of the WRF-Hydro modeling system is described in Gochis et al..

WRF-Hydro-CUFA builds upon the WRF-Hydro system by integrating two components designed for coastal-urban flood applications: (1) a boundary that allows dynamic water levels to be applied along a user-defined coastline, and (2) stormwater drainage modeling via a two-way coupling of WRF-Hydro with SWMM (version 5.1). The latter is achieved by linking the one-dimensional hydraulic flow solver of SWMM with the overland flow routing module of WRF-Hydro using a loosely coupled approach^22^. The SWMM hydraulic engine employs full dynamic wave routing, solving the Saint–Venant equations along conduits to determine net flow rates and ponded water depths at stormwater inlets and outfalls throughout the drainage network. At each coupling interval, the information is exchanged with the overland flow routing module of WRF-Hydro, where it is combined with surface water depths at corresponding grid cells. The updated values are then passed back to SWMM to define the initial and tailwater conditions for solving stormwater drainage flow.

For this study, we implemented WRF-Hydro-CUFA at a 10 m spatial resolution over the Ala Wai Watershed to simulate compound flood inundation in Waikīkī, Hawai‘i (Fig. 1). Expanding the domain to the entire watershed allowed for the inclusion of upstream flood sources, which often contribute to flooding in low-lying areas. Sensitivity testing, aiming to balance numerical stability and computational efficiency, determined a model timestep of 1 h for the Noah-MP LSM and 0.01 s for the terrain routing modules of WRF-Hydro.

Urban drainage in Waikīkī was defined using the hydraulic network developed by Obara et al. (2025). While stormwater drainage networks exist throughout the entire Ala Wai Watershed, this study focused specifically on urban drainage infrastructure within Waikīkī, given its critical role as a conduit for inland floodwaters. As a result, floodwaters generated in areas upstream of Waikīkī that might otherwise be partially mitigated by urban drainage systems are assumed to flow toward and into the Ala Wai Canal, influencing flood patterns in a manner consistent with observed real-world conditions. Additionally, we introduced 30 new inlets and conduits along the eastern side of Waikīkī to enhance drainage capacity. To ensure proper gravity drainage, invert elevations were determined based on nearby drainage infrastructure, maintaining a downslope gradient toward the ocean.

### Model input

Static geographic data was mapped to the model domain using the WRF-Hydro ArcGIS pre-processing toolkit. Topographic elevations were defined from a composite bare earth digital elevation model (DEM), which incorporated data from the University of Hawai‘i School of Earth Sciences and Technology (SOEST), Hawai‘i Mapping Research Group (HMRG), National Centers for Environmental Information (NCEI), NOAA, and USACE. The resulting DEM was corrected for misclassification errors and interpolation artifacts and was provided at a 10 m resolution with a vertical uncertainty of approximately 30 cm. Regional land cover data was obtained from the NOAA Coastal Change Analysis Program (C-CAP) at 2.4 m resolution and remapped to USGS 24-type land cover classes^77,78^. Soil hydraulic parameters^79^ were determined using soil properties from the Soil Survey Geographic Database (SSURGO) at 30 m resolution and remapped to USGS State Soil Geographic (STATSGO) database soil texture classes^80,81^. Based on local observations of a persisting shallow groundwater table that exists a few meters below the surface, we assumed that hydraulic gradients at the bottom of soil columns were negligible.

Dynamic forcing input data was mapped to the model grid using Earth System Modeling Framework (ESMF) re-gridding utilities. Hourly meteorological forcing data was obtained from the Hawai‘i configuration of the Advanced Research Weather Research and Forecasting (HiRES-ARW) model at 1 km resolution. This includes values for incoming shortwave and longwave radiation, specific humidity, air temperature, surface pressure, and near-surface winds. Hourly precipitation forcing was sourced from the Multi-Radar Multi-Sensor System (MRMS) Pass-2 Quantitative Precipitation Estimates (QPEs), also at 1 km resolution.

Coastal water levels were obtained from the NOAA Center for Operational Products and Services (CO-OPS) Honolulu tide gauge (station 1612340) and linearly interpolated to hourly water levels. We assume that uniform water levels from the nearest tide gauge represent the overall tidal conditions along the coastline. When incorporating SLR into coastal boundary conditions, we assume a static increase, adding 0.3 m increments to the coastal boundary dataset.

### Observation data

Observations from monitoring gauges, a pressure transducer deployed in the Ala Wai Canal, and photographic evidence of inland flooding were used to calibrate and validate the flood model. Because traditional flood observations are often limited and costly, the increasing availability of social media imagery provides a valuable modern resource for urban flood detection and assessment^82-83^. In this study, photographic evidence was sourced from local news reports, social media posts, and archived traffic camera snapshots recorded at 15-min intervals. These data highlight the importance of leveraging locally available and non-traditional information sources when direct measurements are sparse.

Canal water levels collected from a pressure transducer in the Ala Wai Canal were used to evaluate model performance in reproducing tailwater conditions influenced by combined rainfall-runoff and tidal forcing. These observations were available for the ULD-2023 and KS-2024 storm events. In addition, water level data from the USGS stream gauge located in the MPDC (station 16247100) upstream of the Ala Wai Canal were used primarily to verify that both tidal signals and precipitation-induced pulses were simultaneously captured during all three storm events. During blue-sky conditions, baseflow contributions were evident in the gauge height measurements. Because the model does not simulate deep subsurface baseflow, tidal peaks observed in the absence of rainfall were assumed to correspond to the imposed tidal boundary conditions. To account for baseflow influence, observed water levels were adjusted by referencing pre-storm conditions to establish a consistent baseline for comparison with simulated results.

Better agreement between simulated and observed canal water levels was achieved at the Ala Wai Canal site (Fig. 3), likely due to its wide, well-defined geometry, which is more accurately captured in the 10 m DEM. In contrast, systematic overestimation of simulated water levels at the MPDC site (Supplementary Fig. 1) is attributed to its narrow channel and dense vegetation cover, which introduces greater uncertainty in channel representation within the DEM.

Additional datasets included observations from the NOAA rain gauge at the Pālolo Fire Station (station 19014414), located upstream of both the Ala Wai Canal and MPDC sites (see Fig. 1 for location), and tidally corrected groundwater levels from a monitoring well in central Waikīkī, which were used to assess simulated subsurface moisture conditions.

### Uncertainties and limitations

Inherent limitations of the WRF-Hydro-CUFA modeling framework arise from its coupling architecture and the physical approximations applied within its hydrologic and hydraulic components. The model uses a diffusive wave formulation for overland flow routing that omits inertial terms in the momentum equations, limiting its ability to represent rapidly varying flows and localized interactions with urban features. Subsurface-drainage exchanges are also not explicitly resolved, as coupling between WRF-Hydro and SWMM is restricted to surface water interactions at inlets and outfalls. A detailed discussion of WRF-Hydro-CUFA development, validation, and associated uncertainties is provided in Son et al. (2023).

Model performance may additionally be influenced by uncertainties in the spatial resolution and quality of input datasets, including the assumption of spatially uniform coastal boundary water levels, and discrepancies between the model grid spacing and the resolution of input data. While higher spatial resolution generally improves the representation of sub-grid topographic features, preliminary testing using a 2 m DEM introduced excessive noise, particularly within canals and streams, restricting inflow and preventing proper tidal signal propagation. These tradeoffs informed the decision to use a 10 m DEM for the final configuration; however, further systematic testing to improve flow pathways at higher resolutions could enhance model accuracy and representation of local hydrodynamics.

In addition to model formulation and input constraints, uncertainties also stem from the limited availability of observational datasets for calibration and validation, and from the lack of comprehensive statistical confidence measures. The scarcity of dense, high-frequency observations across Waikīkī constrains opportunities for rigorous model evaluation and uncertainty quantification. Flood validation in this study is therefore qualitative, relying primarily on photographic and visual documentation rather than spatially continuous measurements. Quantitative comparisons of flood depth and extent were not feasible due to the lack of post-event inundation mapping and high-resolution flood observations. Despite these limitations, integrating multiple data sources, including conventional monitoring networks and non-traditional observations, provides greater confidence in simulated flood patterns. Expanding monitoring networks across Waikīkī and incorporating quantitative validation approaches, such as aerial photogrammetry and flood-depth verification, would substantially improve future model calibration, validation, and flood risk assessments.

## Supplementary Information

Supplementary Information.

## Data Availability

The data that support the findings of this study are available from the corresponding author, K. Y., upon request.
