# Supplementary material for: Compound flood simulations indicate rising public exposure to sewage-contaminated waters in Waikīkī, Hawai‘i
Source: Sci Rep. 2026 Feb 18;16:9740. doi: 10.1038/s41598-026-38225-z (PMC13013977; doi:10.1038/s41598-026-38225-z)
Supplement: Supplementary file 1 — Supplementary Information. [file 41598_2026_38225_MOESM1_ESM.pdf]

Supplementary Information

1. Historical Storms Chosen for this Study

1.1 December 2021 Kona Storm ('KS-2021')

In early December 2021, a strong, slow-moving Kona low situated northwest of the Hawaiian Islands caused widespread flooding, gusty winds, landslides, power outages, road closures, and other storm-related damages. A combination of cold upper-level air and abundant deep moisture from the Tropics created favorable conditions for convective precipitation. The Kona storm event was preceded by a cold front from December 2-4, 2021, that generated locally heavy rainfall across the state but produced only minimal flooding due to its fast-moving nature. Following the frontal passage, the parent upper-low moved southward, pinching off from the jet stream and forming its own circulation as a cutoff low characteristic of Kona storms (National Weather Service, 2021). On December 7, 2021, former Hawaiʻi Governor David Ige declared a state of emergency in response to the ongoing flooding and anticipated severe weather conditions, including heavy rainfall, strong winds, and high surf, which were forecasted to have the potential to cause significant damage to public and private property. Over the next three days, about 10 to 12 inches of rainfall fell on the Waikīkī area characterizing the event as a 50-year storm. In Waikīkī, knee deep floodwaters were reported on Kalākaua Avenue.

1.2 April 2023 Upper-Level Disturbance ('ULD-2023')

On April 1, 2023, an upper-level disturbance brought heavy rainfall and thunderstorms to Oʻahu. The event was marked by unstable atmospheric conditions aloft and a relatively weak surface trough, which generated thunderstorms and heavy downpours, particularly on the south-facing slopes of Oʻahu (National Weather Service, 2023). Flash flood warnings were issued for much of downtown Honolulu, and significant flooding occurred on the H-1 Freeway, leading to road closures and major traffic disruptions. Total rainfall amounts of up to 3 inches were recorded over a 6-hour period, classifying the event as a 5-year storm. Additionally, peak rainfall coincided with the rising tide, further compounding flood impacts.

1.3 May 2024 Kona Storm ('KS-2024')

In early May 2024, a series of late-season disturbances occurred in close succession, bringing heavy rainfall and flooding to many areas of the Hawaiian Islands. Multiple flood watches and warnings were issued across the state, accompanied by reports of localized flooding, road closures, mudslides, vehicle rescues, and other flood damage. On Oʻahu, rain gauges recorded monthly rainfall totals that exceeded twice the May average.

From May 9-12, 2024, a strong low-pressure system aloft and an associated surface trough brought unstable conditions to the state, resulting in episodes of heavy rainfall and flooding. On May 11, 2024, the system shifted westward over Oʻahu, causing periods of rain showers focused on the central and western areas of the island. Although the Ala Wai Watershed was affected, daily precipitation totals for Honolulu were insignificant. In the following days, low-level east-southeasterly flow from the system interacted with the island terrain, leading to continued heavy downpours and severe flooding on the windward side of Oʻahu. By May 14-15, 2024, stable conditions developed as the low-pressure system aloft weakened. Meanwhile, a Kona low system developed north of Kauaʻi and gained strength as it moved westward. Hawaiʻi Governor Josh Green signed an Emergency Proclamation in preparation for the Kona storm, in part due to the wet conditions produced beforehand. On May 16, 2024, the main rain band settled over Oʻahu, causing periods of intense rainfall and flooding for the next 24 to 36 hours. In addition, elevated water levels along south-facing shores were reported due to overlapping, long period south-southwest swells, causing a High Surf Advisory, with a threshold of 1-ft, to remain in effect during the storm (National Weather Service, 2024).

2. Defining Precipitation Frequency

Point Precipitation Frequency Estimates (PFEs) for Waikīkī were provided by the NOAA Atlas-14, Volume 4, Version 3 data available online through the precipitation frequency data server. Partial duration time series data for precipitation depth was selected for the USGS WAIKIKI 717.2 (51-9397) station. For the three storm events, we referenced this data together with values of accumulated precipitation over storm durations from Pass-2 Multi Radar Multi-Sensor System (MRMS) Quantitative Precipitation Estimation (QPE) accumulation values, to define the return period of each storm.

3. Supplementary Figure

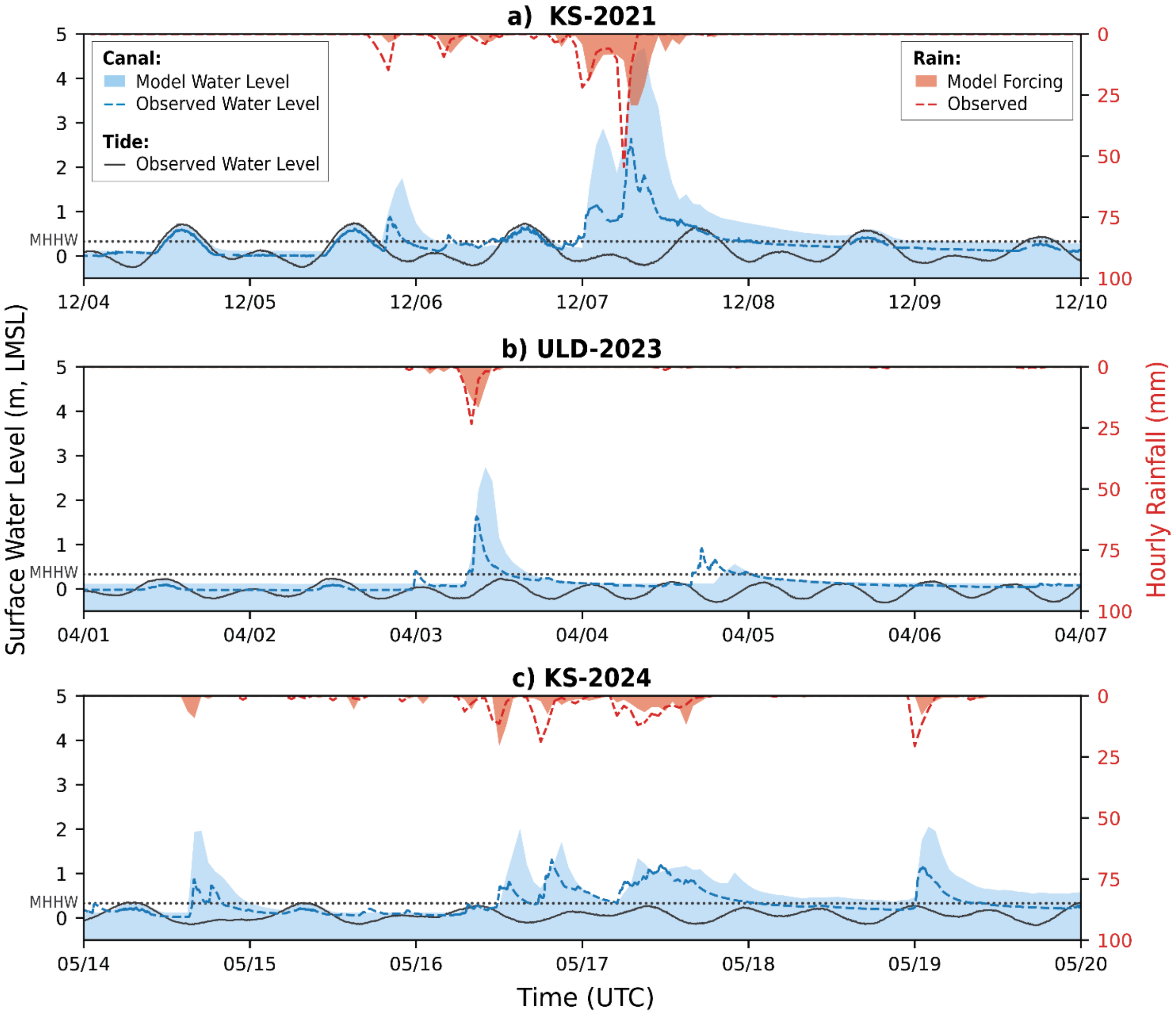

Supplementary Figure 1. *Simulated (shaded blue) and observed (dashed blue) water levels at the Mānoa-Pālolo Drainage Canal (MPDC) upstream of Waikīkī during (a) KS-2021, (b) ULD-2023, and (c) KS-2024. Tide observations from the Honolulu gauge (solid black) and the Mean Higher High Water (MHHW; dotted black) are shown for reference. The upper portion of each panel shows hourly rainfall forcing (shaded red) and observed rainfall (dashed red) at the location of the Pālolo Fire Station rain gauge. The plots highlight how variations in rainfall and tides influence floodwater behavior and demonstrate the model's ability to capture these interactions, emphasizing the importance of accounting for compound processes in such environments.*
